# Supplementary material for: Predicting Invasiveness in Lepidic Pattern Adenocarcinoma of Lung: Analysis of Visual Semantic and Radiomic Features
Source: Med Sci (Basel). 2024 Oct 18;12(4):57. doi: 10.3390/medsci12040057 (PMC11503399; doi:10.3390/medsci12040057)
Supplement: Supplementary file 1 [file medsci-12-00057-s001.zip › medsci-3197827-supplementary.pdf]

medical sciences

Supplementary materials:

Table S1. Radiomic texture features.

Histogram (11 variables)	tumor_stddev, tumor_perc95, tumor_perc90, tumor_perc25, tumor_perc20, tumor_perc15, tumor_perc10, tumor_perc05, tumor_median, tumor_kurtosis, mean_HU
Size (4 variables)	volume, perpendicular diameter, diameter in axial plane, longest diameter including z-axis
GLCM* (80 variables)	glcm_variance_8, glcm_variance_64, glcm_variance_32, glcm_variance_16, glcm_sum_variance_8, glcm_sum_variance_64, glcm_sum_variance_32, glcm_sum_variance_16, glcm_sum_entropy_8, glcm_sum_entropy_64, glcm_sum_entropy_32glcm_sum_entropy_16, glcm_sum_average_8, glcm_sum_average_64, glcm_sum_average_32, glcm_sum_average_16, glcm_min_8, glcm_min_64, glcm_min_32, glcm_min_16, glcm_mean_8, glcm_mean_64, glcm_mean_32, glcm_mean_16, glcm_max_corr_coef_8, glcm_max_corr_coef_64, glcm_max_corr_coef_32, glcm_max_corr_coef_16, glcm_max_8, glcm_max_64, glcm_max_32, glcm_max_16, glcm_info_corr_b_8, glcm_info_corr_b_64, glcm_info_corr_b_32, glcm_info_corr_b_16, glcm_info_corr_a_8, glcm_info_corr_a_64, glcm_info_corr_a_32, glcm_info_corr_a_16, glcm_homogeneity_8, glcm_homogeneity_64, glcm_homogeneity_32, glcm_homogeneity_16, glcm_entropy_8, glcm_entropy_64, glcm_entropy_32, glcm_entropy_16, glcm_energy_8, glcm_energy_64, glcm_energy_32, glcm_energy_16, glcm_dissimilarity_8, glcm_dissimilarity_64, glcm_dissimilarity_32, glcm_dissimilarity_16, glcm_diff_variance_8, glcm_diff_variance_64, glcm_diff_variance_32, glcm_diff_variance_16, glcm_diff_entropy_8, glcm_diff_entropy_64, glcm_diff_entropy_32, glcm_diff_entropy_16, glcm_diff_average_8, glcm_diff_average_64, glcm_diff_average_32, glcm_diff_average_16, glcm_correlation_8, glcm_correlation_64, glcm_crrelation_32, glcm_correlation_16, glcm_contrast_8, glcm_contrast_64, glcm_contrast_32, glcm_contrast_16, glcm_angular_2nd_moment_8, glcm_angular_2nd_moment_64, glcm_angular_2nd_moment_32, glcm_angular_2nd_moment_16

glcm_name_N*, where glcm is abbreviation of gray level co-occurrence matrix, name is the feature, and N is the number of discrete intensity levels in the lesion. Here, we used 8, 16, 32, and 64 discretized intensity levels.

Table S2. Summary statistics for radiomic variables.

	N=33							
	Invasive (n=24)				Non-Invasive (n=9)			
	mean	sd	min	max	mean	sd	min	max
histogram data								
tumor_stddev	188.0	35.7	118.1	253.0	163.8	40.3	105.2	241.1
tumor_perc95	-84.4	138.0	-377.1	92	-184.0	152.4	-398	25
tumor_perc90	-153.4	162.1	-476	58	-256.2	160.9	-477	-23.7
tumor_perc25	-549.6	139.7	-793	-256	-607.8	97.3	-732	-443
tumor_perc20	-577.6	128.6	-816	-317	-626.4	95.0	-741	-466
tumor_perc15	-607.4	119.5	-847	-382	-646.1	95.1	-768	-487
tumor_perc10	-638.8	110.8	-887	-460.2	-668.8	96.9	-815.9	-507.7
tumor_perc05	-685.0	99.9	-941	-507	-705.7	108.0	-881	-533.6
tumor_median	-420.6	188.2	-734	-58	-509.7	121.3	-675	-304.5
tumor_kurtosis	0.12	1.05	-1.14	2.39	0.29	1.16	-1.13	2.14
mean_HU	-408.5	153.1	-701.2	-151.3	-484.3	119.9	-647.0	-288.8
Size								
perp_diameter	18.9	11.6	4.0	52.9	14.5	5.5	6.8	25.9
diameter	25.4	15.6	7.1	78.0	19.3	6.9	8.3	28.5
dia_z	51.1	55.8	10	219	41.4	15.8	12	64
volume	7331.1	10203.4	314.9	43854.7	2095.4	1646.3	286.2	5284.8
feature								
glcm_correlation_64	0.59	0.12	0.35	0.78	0.53	0.14	0.26	0.74
